# The Role of Small Bowel Capsule Endoscopy in Determining the Treatment Strategy for Duodenal Follicular Lymphoma: A Single-Center Retrospective Study

**DOI:** 10.3390/diagnostics15020193

**Published:** 2025-01-15

**Authors:** Donghoon Kang, Gi-June Min, Tong Yoon Kim, Young-Woo Jeon, Yukyung Cho, Jae Myung Park, Joo Hyun O, Byung-Ock Choi, Gyeongsin Park, Seok-Goo Cho

**Affiliations:** 1 Department of Gastroenterology and Hepatology, Catholic University Lymphoma Group, Seoul St. Mary’s Hospital, College of Medicine, The Catholic University of Korea, Banpo-daero 222, Seocho-Gu, Seoul 06591, Republic of Korea; etiria@gmail.com (D.K.); ykcho@catholic.ac.kr (Y.C.); parkjerry@catholic.ac.kr (J.M.P.); 2 Department of Hematology, Catholic University Lymphoma Group, Seoul St. Mary’s Hospital, College of Medicine, The Catholic University of Korea, Banpo-daero 222, Seocho-Gu, Seoul 06591, Republic of Korea; beichest@catholic.ac.kr; 3 Department of Hematology, Catholic University Lymphoma Group, Yeouido St. Mary’s Hospital, College of Medicine, The Catholic University of Korea, 10 63-ro, Yeongdeungpo-gu, Seoul 07345, Republic of Korea; tyk@catholic.ac.kr (T.Y.K.); native47@catholic.ac.kr (Y.-W.J.); 4 Department of Nuclear Medicine, Catholic University Lymphoma Group, Seoul St. Mary’s Hospital, College of Medicine, The Catholic University of Korea, Banpo-daero 222, Seocho-Gu, Seoul 06591, Republic of Korea; ojoohyun@songeui.ac.kr; 5 Department of Radiation Oncology, Catholic University Lymphoma Group, Seoul St. Mary’s Hospital, College of Medicine, The Catholic University of Korea, Banpo-daero 222, Seocho-Gu, Seoul 06591, Republic of Korea; choibo67@catholic.ac.kr; 6 Department of Hospital Pathology, Catholic University Lymphoma Group, Seoul St. Mary’s Hospital, College of Medicine, The Catholic University of Korea, Banpo-daero 222, Seocho-Gu, Seoul 06591, Republic of Korea; gspark@catholic.ac.kr

**Keywords:** duodenal follicular lymphoma, video capsule endoscopy, small bowel, chemotherapy

## Abstract

**Objectives**: In this single-center retrospective study, we aimed to verify the extent of duodenal follicular lymphoma (DFL) and investigate the role and clinical significance of video capsule endoscopy (VCE) in the treatment process. **Methods**: We analyzed the clinical and imaging data of 40 patients diagnosed with DFL. **Results**: Imaging workup and bone marrow biopsies revealed DFL only in the gastrointestinal tract (stage I) in 22 patients and in local lymph nodes (stage II_1_), distant lymph nodes (stage II_2_), pancreas (stage II_2_E_pancreas_), and extranodal regions (stage IV) in 1, 3, 1, and 13 patients, respectively. Fifteen of the 23 patients with localized (stages I and II_1_) DFL underwent VCE for comprehensive small bowel evaluation, which revealed lesion extension beyond the duodenum in 10 patients (66.7%). A watch-and-wait strategy was implemented for one patient and systemic chemotherapy was administered to the remaining nine. Of the eight patients without VCE, seven and one received radiotherapy and observation, respectively. Nine of the 23 patients (39.1%) received systemic treatment based on positive VCE results. Only one of the 17 patients with advanced-stage DFL (stages II_2_ and IV) accepted radiotherapy; 16 underwent systemic chemotherapy. During follow-up (median, 48.4 months), two relapse events occurred in the advanced stage, with no lymphoma-associated deaths. DFL tends to be indolent and has favorable outcomes. **Conclusions**: Proactive VCE for diagnosing DFL is recommended to determine small bowel involvement, which may influence subsequent treatment decisions.

## 1. Introduction

Duodenal-type follicular lymphoma (DFL) is recognized as a distinct variant within the spectrum of follicular lymphomas (FL) and is incidentally diagnosed during esophagogastroduodenoscopy (EGD) owing to its relatively low incidence and frequently asymptomatic nature. Due to the high prevalence of gastric cancer, a national cancer screening program (which includes biannual EGD examinations) has been in operation in Korea for 40 years. Gastrointestinal FL, particularly DFL, is readily accessible for diagnosis through endoscopy, making it well-suited for detection in screening programs. The most recent edition of the World Health Organization (WHO) classification of lymphoid neoplasms recognizes DFL as a distinct entity separate from other gastrointestinal tract FLs [1]. DFL manifests as white nodular lesions on the second portion of the duodenum during endoscopic examinations, whereas nodal follicular lymphoma (NFL) often presents as extrinsic lesions causing lumen compression. Additionally, DFL is characterized by a lower histological grade (grade 1/2) and shares many features with in situ follicular neoplasia that are associated with favorable outcomes.

DFL typically progresses slowly and has minimal impact on patients’ quality of life. Therefore, similar to classical FL with nodal disease, there remains debate regarding the need for treatment of patients with DFL. While consensus has not been reached, the most commonly employed approach is the watch-and-wait strategy, where active treatment is deferred until there is clear evidence of disease progression or manifestation of symptoms [2,3]. Due to its localization and gene expression profile, DFL shares greater similarity with mucosa-associated lymphoid tissue lymphoma than NFL [1]. Therefore, radiation therapy is the first-line treatment for newly diagnosed DFL at many institutes where irradiation is a viable option for localized indolent lymphoma.

Several previous studies have reported that DFL is primarily located in the duodenum, although it can sometimes extend to the distal small intestine with or without other extranodal lesions [4,5]. In 2011, a multicenter retrospective study conducted in Japan analyzed 125 patients with stage I and II gastrointestinal FL [6]. Among these patients, 70 underwent double-balloon enteroscopy (DBE) and/or video capsule endoscopy (VCE), and the most frequent place where a lesion was located was in the second portion of the duodenum, followed by the jejunum, whereas 85% of the patients had lesions involving the duodenum and extended to the jejunum or ileum [6].

Therefore, this study aimed to verify the extent of DFL and investigate the role and clinical significance of VCE in this process. The main objective was to assess the presence of small bowel involvement in patients diagnosed with DFL and to compare two groups: those with small bowel extension beyond the duodenum and those where lymphoma was confined solely to the duodenum before receiving appropriate treatment.

## 2. Materials and Methods

### 2.1. Ethics Approval

The study protocol was approved by the Institutional Review Board and Ethics Committee of the Catholic Medical Center, Republic of Korea (approval number: KC16OISI0771), and was conducted in accordance with the tenets of the Helsinki Declaration. This research involved no more than minimal risk; due to the necessity of a waiver of informed consent, obtaining consent from participants was not applicable to this study. The waiver of informed consent was granted without adverse impact on the rights and welfare of the study participants and was approved by the Institutional Review Board of the Catholic Medical Center, Republic of Korea.

### 2.2. Study Design, Patient Selection, and Diagnosis

In this single-center retrospective study, we reviewed the medical records of 40 patients diagnosed with DFL at Seoul St. Mary’s Hospital between January 2015 and December 2022 and analyzed patients’ clinical features and treatment outcomes. All enrolled patients were managed by the Catholic University Lymphoma Group, a multidisciplinary team comprising expert hematologists, pulmonologists, hematopathologists, radiologists, and nuclear medicine specialists. The medical records were thoroughly reviewed to obtain data on patient demographics, laboratory test values, date of diagnosis, treatment modality, and last follow-up date for clinical prognosis. Well-experienced gastroenterologists performed EGD and biopsy for all patients. DFL was diagnosed based on the endoscopic findings for white and nodular lesions in the duodenum and on the pathological report provided by two independent expert pathologists who confirmed the pathological diagnosis of DFL based on the morphologic and immunophenotypic features defined according to the 2022 WHO classification [1].

### 2.3. Evaluation of Study Patients

**Figure 1:**
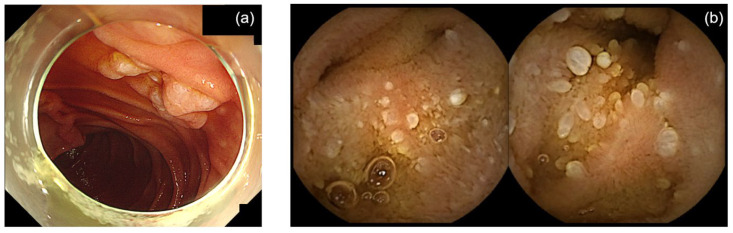
Duodenal-type follicular lymphoma involving distal jejunum. (**a**) Duodenal follicular lymphoma diagnosed using upper endoscopy. (**b**) Small bowel involvement in duodenal-type follicular lymphoma in the jejunum diagnosed using video capsule endoscopy.

DFL staging was based on physical examination, laboratory studies, and radiological findings, including neck, chest, and abdomen/pelvis imaging using computed tomography (CT), and on fluorodeoxyglucose-positron emission tomography/CT (FDG-PET/CT) based on the Lugano classification for gastrointestinal tract lymphoma (Supplementary Table S1) [7,8]. DFL can be staged from stages I to IV depending on single or multiple gastrointestinal tract involvement, degree of intestinal wall infiltration, secondary lymph node involvement of adjacent or distant organs, or disseminated commitment. If lymphoma exists within the abdominal layer, a single primary lesion (mostly in the second portion of the duodenum) or multiple non-continuous extensions of the lesions are indicative of stage I. Stages II_1_ and II_2_ indicate that the lymphoma extends into local and distant nodal sites, respectively. Stage IIE indicates penetration of the lymphoma to the serosa involving adjacent organs or tissues without invading distant organs, whereas stage IV indicates disseminated extranodal involvement of the gastrointestinal tract with supra-diaphragmatic nodal involvement. BM biopsy was performed to confirm BM involvement; VCE was performed to detect the jejunal or ilial spread of the DFL, which could not be detected on imaging (Figure 1 and Figure S1). Treatment decisions were based on the stage, which was categorized as localized (stages I and II_1_) or advanced (stages II_2_ and IV). The prognostic group was stratified according to the Follicular Lymphoma International Prognostic Index [9]. Follow-up data included endoscopic findings, pathological data, and details of the treatments administered by the patients.

### 2.4. Treatment and Response Evaluation

Chemotherapy was administered if small intestinal DFL spread was evident, either on imaging or VCE, even in cases at the localized stage. Moreover, chemotherapy was administered to patients at advanced disease stages with evidence of disseminated nodal or extranodal involvement. The primary systemic chemotherapy regimen comprised cyclophosphamide (750 mg/m^2^ on day 1), vincristine (1.4 mg/m^2^ on day 1), and prednisone (60 mg/m^2^ on days 1–5) combined with rituximab (375 mg/m^2^ on day 1). Bendamustine (90 mg/m^2^ on days 1–2) and rituximab (375 mg/m^2^ on day 1), known as the BR regimen, have been administered since 2018, and their costs were not covered by health insurance in Korea and were incurred by the patients. The patients underwent involved-site radiotherapy (RT) with curative intent only when the DFL lesion was localized to the duodenum. The clinical target volume included the entire duodenum and was prescribed at a dose of 24 Gy in 12 fractions. The internal target volume and planning target volume were determined using the motion information obtained from the four-dimensional CT to assess breathing motions. They were defined as an expansion of 5 mm from the internal target volume, considering the set-up error of the patient. Patients with DFL extending beyond the duodenum were excluded from RT.

Surveillance for therapeutic response and monitoring of local or systemic relapse was routinely conducted using FDG-PET/CT. Neck, chest, abdominal, and pelvic CT were performed using EGD, and laboratory testing was performed every 6 months for a period of over 5 years. The radiological response was categorized based on the Lugano classification as follows: (i) complete response (CR), which is defined as no macroscopic tumor and negative histopathologic findings; (ii) partial response, which is defined as at least 50% reduction in the macroscopic tumor and its corresponding histopathologic findings; (iii) stable disease (SD), which is defined as minimal changes within 25% of the macroscopic mass with positive histopathologic findings; or (iv) progressive disease (PD), which is defined as worsening macroscopic findings [7,8].

### 2.5. Statistical Analyses

All categorical variables were compared using the Chi-squared or Fisher’s exact test. Student’s *t*-test and Mann–Whitney U test were performed to compare continuous variables between the two groups. The primary endpoint of this study was progression-free survival (PFS), defined as the time from diagnosis to relapse after achieving CR, PD despite administering treatment, or death due to any causes. Overall survival (OS) was defined as the time from diagnosis to death. PFS and OS were estimated using the Kaplan–Meier method and compared using the log-rank test. Cumulative incidence of relapse (CIR) was calculated using cumulative incidence estimation, and non-relapse mortality was considered a competing risk. Significance was determined at a two-tailed *p*-value < 0.05. All statistical analyses were performed using SPSS (version 13.0; SPSS Inc., Chicago, IL, USA) and R software (version 4.3.1; R Foundation for Statistical Computing, Vienna, Austria, 2023).

## 3. Results

### 3.1. Initial Evaluation

Forty patients diagnosed with DFL at Seoul St. Mary’s Hospital between 2015 and 2022 were enrolled and analyzed. The baseline characteristics of the patients are summarized in Table 1. The median age of the patients was 49.5 years (range, 36–77 years), with female predominance (men, 37.5% and women, 62.5%). Due to the limited accessibility to VCE, imaging studies and BM biopsies were primarily performed. Of the 40 patients, 22 showed lymphoma only in the duodenum with or without extension to the small bowel (stage I), 1 had local nodal involvement (stage II_1_), 3 had distant nodal involvement in the abdomen (stage II_2_), 1 had pancreatic involvement (stage II_2_E_pancreas_), and 13 had extranodal involvement (stage IV). Among the 17 patients with advanced stages of the disease (stage II_2_ and IV), extended involvement of the lymph nodes was observed in 8 patients, distant organ involvement in 2, and bone marrow (BM) involvement in 7. Based on the imaging study and BM biopsy, 23 patients were classified as having localized-stage disease (stages I and II_1_) and could be candidates for the wait-and-watch approach. A comparison of clinical characteristics and treatment modalities between patients with localized and advanced-stage disease is presented in Supplementary Table S2.

### 3.2. Small Bowel Involvement in Patients with DFL

**Figure 2:**
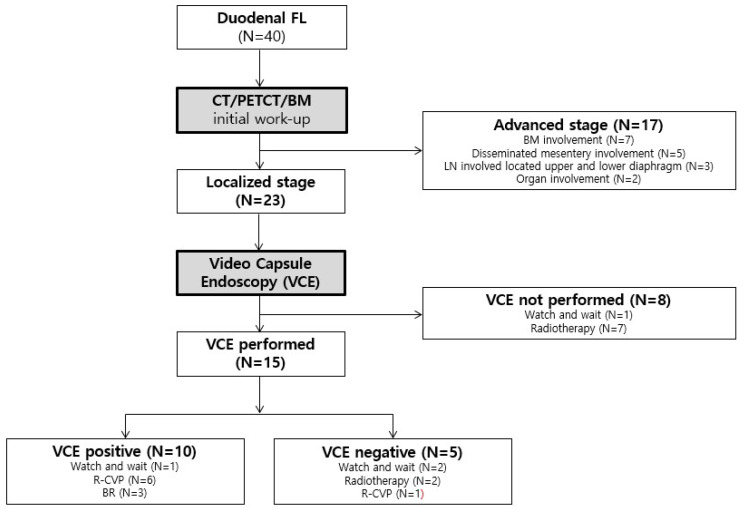
CONSORT flowchart of the treatment algorithms of the enrolled patients with DFL for either localized or advanced stage (N = 40). BM, bone marrow; DFL, duodenal follicular lymphoma; R-CVP, cyclophosphamide, vincristine, and prednisone combined with rituximab; VCE, video capsule endoscopy; CT, computed tomography, PET/CT, fluorodeoxyglucose-positron emission tomography/CT; and LN, lymph node § among 17 advanced stage DLF patients.

Among the 23 patients with localized-stage DFL, 15 (65.2%) underwent VCE for thorough evaluation of the small intestine, 10 out of these 15 (66.7%) exhibited small bowel extension and 5 (33.3%) did not show any extension beyond the duodenum. The majority of patients who were confirmed to have small bowel extension received systemic chemotherapy; however, some patients did not receive any treatment, which was at their discretion. In contrast, among the patients who showed negative VCE findings in the localized stage, two received RT and one received chemotherapy (Stage II_1_), whereas two patients were placed on the wait-and-watch approach. Meanwhile, 12 of the 17 patients (70.6%) with advanced-stage DFL underwent VCE, and 9/12 (75.0%) patients presented with small bowel involvement. All patients with advanced-stage DFL received chemotherapy, except for one patient who opted for RT after declining chemotherapy. The treatment algorithms for localized or advanced stages of DFL are shown in Figure 2. Overall, 27 patients, either with localized or advanced stage, underwent VCE, and 19/27 patients (70.4%) showed positive findings, indicating that the DFL extended through the small bowel. Table 2 presents a comparison of clinical characteristics between the patients who showed small bowel involvement and those who showed negative findings after VCE.

### 3.3. Treatment Modalities and Responses

Among the 23 patients with localized DFL, 10 patients in the chemotherapy group showed long-term CR without relapse (median, 62.9 and range, 12.6–81.9 months). All eight patients who underwent RT also achieved CR (median, 50.1 and range 12.4–96.2 months), but one patient died due to causes other than lymphoma. The four patients with DFL who decided to adopt the wait-and-watch strategy remained alive (median, 41.5 and range, 26.7–66.0 months). However, all patients in the wait-and-watch group underwent routine check-ups with EGD, VCE, and imaging studies within 3–6 months until PD. In addition, 9/40 patients (22.5%) changed their treatment strategy, while 1/40 (2.5%) refused to change based on the results of VCE. Among the 16/17 patients with advanced stage who were administered chemotherapy, 15 patients achieved CR (median, 29.3 and range, 13.1–75.6 months), whereas the patient who received rituximab monotherapy showed PD of 12.2 months after achieving remission, received six cycles of the BR salvage regimen, and finally achieved CR. One patient received involved-field RT in the second portion of the duodenum; despite showing low maximum standard unit value (SUVmax), multifocal mesenteric uptake was observed on FDG-PET/CT and the case was considered stage IV. The patient refused to receive combined chemotherapy and eventually showed PD of 10.1 months after RT. After PD, the patient received six cycles of the BR salvage regimen and achieved CR.

**Figure 3:**
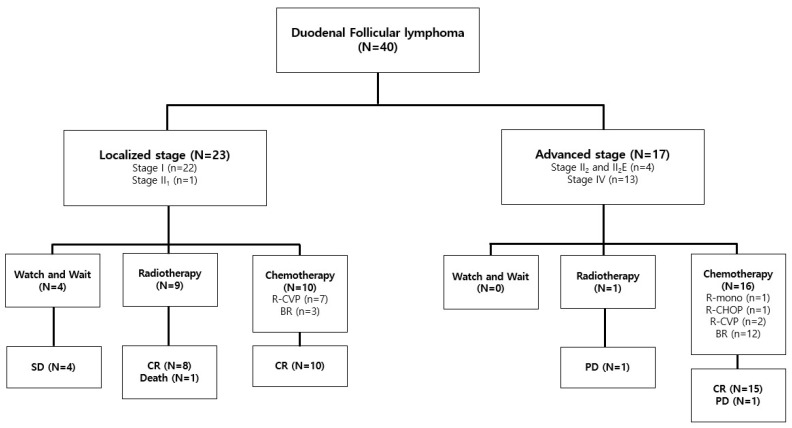
Treatment outcomes of patients with DFL (N = 40). BR, bendamustine–rituximab; CR, complete response; PD, progressive disease; R-CHOP, cyclophosphamide, doxorubicin, vincristine, and prednisone combined with rituximab; R-CVP, cyclophosphamide, vincristine, and prednisone combined with rituximab; R-mono, rituximab monotherapy; and SD, stable disease.

In the median follow-up duration of 48.4 months (range, 12.4–96.2), OS and PFS were 94% (95% confidence interval [CI], 68.1–99.2) and 89.4% (95% CI, 68.0–96.8), respectively, with a CIR of 5.0% (95% CI, 0.9–15.0). One death event was attributed to ampulla of Vater disease progression, and not to lymphoma. Two PD and one death event were identified as PFS and CIR events, respectively. The treatment outcomes of the patients with DFL are summarized in Figure 3; the detailed clinical courses of seven patients who showed relapse, died, or showed outcomes of the watch-and-wait strategy for DFL are summarized in Table 3.

## 4. Discussion

In our study, we verified the extent of DFL and investigated the role and clinical significance of VCE. An extended lesion in the small bowel was observed in approximately 70% (19/27) of the patients who underwent VCE, similar to the findings of a previous study, which showed a high incidence (85%) of detected lesions in other regions of the small bowel, primarily the jejunum [4,6]. Owing to the indolent nature and slow-growing characteristics of DFL, the wait-and-watch approach is regarded as an acceptable alternative for patients with DFL. A comparison between rituximab combined with chemotherapy and the wait-and-watch approach in patients with intestinal FL revealed similar outcomes [5,10]. This finding supports the suitability of the wait-and-watch approach for managing DFL. Further, another study observed cases of spontaneous shrinkage or disappearance of gastrointestinal FL [11]. RT may be considered an alternative approach if the lesion is limited to the radiation field [5]. However, it is essential to conduct a thorough evaluation using colonoscopy and small intestinal endoscopy before deciding to implement RT, as DFL often involves other parts of the intestines and DFL in distant lesions is not always perceptible by FDG-PET/CT or CT [12], as reported in our findings.

Nevertheless, if DFL is considered an early lesion, consistent with the concept of in situ follicular neoplasia, similar rates of progression can be anticipated in both conditions. However, owing to the rarity of the disease and the limited number of available studies, an accurate estimation of these rates is currently unavailable. Recent research has emphasized that the circumferential location and fusion of follicular lesions are significant predictors of disease progression or transformation into diffuse large B-cell lymphoma [13]. Patients with these endoscopic features may require short-term surveillance for close monitoring of their condition. In addition, a previous study examined the wait-and-watch strategy in 33 patients with gastrointestinal FL, the majority of which were located in the duodenum and categorized as grade 1 according to the WHO pathological criteria. They reported instances of disease progression and transformation to diffuse large B-cell lymphoma [10].

Among the available treatment approaches for DFL, the wait-and-watch approach does not require the administration of immediate treatment. However, as mentioned earlier, there is a possibility of high-grade transformation necessitating continuous follow-up examinations [10,14]. Consequently, routine surveillance tests such as routine endoscopy, VCE, CT, and FDG-PET/CT are essential. The lack of a clear consensus on the duration of follow-up increases the burden on patients both psychologically and economically, as they may have to undergo continuous tests without knowing when the tests can be discontinued [15]. Although RT can be a viable alternative [16], the involvement of distant small bowel, as observed in this study, was reported in a considerable number of cases. Thus, it is crucial to select patients carefully and conduct a thorough evaluation, including VCE at the initial diagnostic stage. In our study group, seven patients underwent RT without evidence of VCE and did not experience a relapse, but this practice may not be justified because of the potential involvement of the small bowel.

Systemic chemotherapy based on rituximab can be considered the most definitive treatment alternative, given its highly evident therapeutic effects [17]. Moreover, compared to NFL, a reduced presence of effector regulatory T cells within neoplastic follicles was noted in DFL, which could explain the stronger antitumor responses observed. This effect is likely due to the increased infiltration and activity of CD8+ tumor-infiltrating lymphocytes, resulting in a more indolent clinical profile in DFL [18]. However, due to the possibility of occurrence of various systemic side effects associated with systemic chemotherapy, appropriate patient groups should be selected for administration. The overall outcomes of DFL in this study were outstanding, with only two relapse events occurring in patients in the advanced-stage group. One patient was treated with rituximab monotherapy for Stage II_2_E_pancreas_. The other patient received involved-field RT in the second portion of the duodenum; however, multifocal mesenteric uptake was observed on FDG-PET/CT despite the low SUVmax values, and the lesion was considered a stage IV lesion. One death event was reported in this cohort, but the patient died from ampulla of the Vater cancer progression, and not lymphoma.

In this study, most patients with confirmed nodal and extranodal involvement based on initial CT and FDG-PET/CT received systemic chemotherapy as the primary treatment. However, for patients with negative findings on these imaging examinations but with lesions confined to the second portion of the duodenum, further evaluation of the extent of small bowel involvement requires VCE or DBE. Nevertheless, due to the limited access to VCE and DBE, it is advisable to select eligible patients for these procedures, if feasible. We routinely administered systemic chemotherapy to enhance clinical outcomes, particularly in patients with stage I with extended lymphoma involvement in the small bowel, as confirmed by VCE. Approximately 43.5% of the patients with localized-stage underwent systemic chemotherapy as a first-line treatment. The risks and benefits of chemotherapy compared to those of the wait-and-watch strategy, which is globally accepted for indolent lymphoma, were discussed. Thus, post-chemotherapy follow-up will be based on the remission state of the disease, and much less frequent workup would be necessary. Moreover, if the disease progresses, there would be a high risk of intestinal perforation or other complications after the late administration of chemotherapy. Given that DFL makes it challenging to evaluate the condition based on imaging and symptoms alone, if high-grade transformation or duodenal bleeding occurs, patients may experience a catastrophic situation. However, in our study, no significant clinical differences were observed between patients with and without involvement of the lower part of the small bowel, as confirmed by VCE, and an aggressive treatment approach for DFL might not be fully justified in patients with stage I DFL.

The WHO distinguished DFL from existing gastrointestinal FL following the revised classification of lymphoid neoplasms in 2016 [19]. Previous studies on DFL focused on lesions involving the duodenum as part of the research on gastrointestinal FL. The invasion of the second portion of the duodenum by conventional gastrointestinal FL is a negative risk factor. Notably, these studies were not limited to patients with DFL. However, our study had the advantage of focusing exclusively on patients with pathologically confirmed DFL and benefitted from the inclusion of a relatively large cohort of 40 patients from the same institution, who were all treated using the same treatment methodologies. To the best of our knowledge, this is the first study to compare patients who underwent VCE to confirm the presence of lesions with those who did not. This study highlights the need for proactive VCE at the initial diagnostic stage, given the lack of precise biomarkers for suspected proximal small bowel involvement before VCE is performed.

This study has some limitations. First was its retrospective, single-center design. Second, given the indolent nature of DFL with there being a long time-to-event, relatively rare events occurred in many censored patients, which may have introduced some bias and rendered it difficult to statistically show the treatment effect. Third, multivariate analysis could not be performed. At our institute, we aggressively use systemic chemotherapy, if indicated, for better clinical outcomes, even for indolent lymphoma, including DFL. However, given the relatively low incidence of DFL, the analysis focused on patients only diagnosed with pathologically confirmed DFL, which adds significance to this study. Our findings can be further confirmed if sufficient data can be obtained from a larger number of patients in the future.

Additionally, there is no definitive data regarding the sensitivity and specificity of VCE in detecting intestinal lymphoma. However, earlier studies on first- and second-generation VCE reported a detection rate of 55.9% (range: 46.0–65.6%) for neoplastic lesions in the small intestine [20]. More recently, AI-assisted autonomous detection demonstrated over 95% sensitivity, specificity, and accuracy in similar applications [21]. The integration of AI into VCE could significantly enhance its diagnostic performance, making it a promising tool for DFL diagnosis and treatment.

## 5. Conclusions

DFL tends to be an indolent disease with favorable outcomes. When diagnosing DFL, it is crucial to proactively conduct VCE to determine small bowel involvement. This information can be used to guide treatment decisions. Given that the disease stage and initial treatment responses are associated with lower PFS, large-scale multicenter studies are required to further evaluate the appropriate therapeutic modalities for DFL and its associated prognostic factors.

## Figures and Tables

**Table 1:** Clinical characteristics of patients with duodenal follicular lymphoma (*n* = 40).

Characteristics	Values
Age (years), median (range)	49.5 (36–77)
Age >60	6 (15.0%)
Sex	
Male	15 (37.5%)
Female	25 (62.5%)
Hemoglobin (g/dL), median (range)	14.0 (8.5–16.8)
≥12	33 (82.5%)
<12	7 (17.5%)
Lactate dehydrogenase (IU/L), median (range)	305.5 (131–612)
Normal	36 (90.0%)
Elevated	4 (10.0%)
Nodal involvement	
0–4	32 (80.0%)
>4	8 (20.0%)
Bone marrow involvement	
No	33 (82.5%)
Yes	7 (17.5%)
Initial VCE	
No	13 (32.5%)
Yes	27 (67.5%)
VCE findings	N = 27
No findings	8 (29.6%)
Extended to proximal jejunum	3 (11.1%)
Extended to distal jejunum	5 (18.5%)
Distal jejunum to ileum	1 (3.7%)
Extended through entire small bowel	10 (37.1%)
Pathologic grade	
Grade 1	30 (75.0%)
Grade 2	10 (25.0%)
Lugano stage	
Localized stage	N = 23
Stage I	22 (55.0%)
Stage II_1_ (local nodal involvements, para-intestinal)	1 (2.5%)
Advanced stage	N = 17
Stage II_2_ (distant nodal involvement, intra-abdomen)	3 (7.5%)
Stage II_2_E (adjacent organ involvement) §	1 (2.5%)
Stage IV †	13 (32.5%)
FLIPI score	
Low risk	29 (72.5%)
Intermediate risk	9 (22.5%)
High risk	2 (5.0%)
Outcome at the final visit	
Alive with lymphoma	4 (10.0%)
Alive, remission state after first-line treatment	33 (82.5%)
Relapsed, alive, remission state after salvage treatment	2 (5.0%)
Dead by other causes than lymphoma	1 (2.5%)

FLIPI, Follicular Lymphoma International Prognostic Index; IU, international units; and VCE, video capsule endoscopy. § Patients with stage II_2_E_[pancreas]_ presented distant nodal and adjacent organ involvement. † Two patients with Lugano stage IV, classified as Ann Arbor stage III, showed both GI tract and multifocal supra-diaphragmatic nodal involvement.

**Table 2:** Comparison of clinical characteristics between patients with negative video capsule endoscopy findings and those with positive small bowel involvement (N = 27).

	VCE Negative (N = 8)	VCE Positive (N = 19)	*p*-Value
Age > 60 years	1 (12.5%)	3 (15.8%)	1.000
Male/Female (n, %)	2 (25.0%)/6 (75.0%)	8 (42.1%)/11 (57.9%)	0.666
Hemoglobin <12.0 (g/dL)	1 (12.5%)	4 (21.1%)	1.000
Elevated LDH (U/L)	1 (12.5%)	1 (5.3%)	0.513
Nodal areas involvement >4	1 (12.5%)	3 (15.8%)	1.000
Extranodal involvement ≥2	1 (12.5%)	4 (21.1%)	1.000
Bone marrow involvement	0 (0%)	5 (26.3%)	0.108
Lugano stage			
I	4 (50.0%)	10 (52.6%)	1.000
II_1_	1 (12.5%)	0 (0%)	0.296
II_2_	0 (0%)	2 (10.5%)	1.000
IV	3 (37.5%)	7 (36.8%)	0.974
FLIPI risk stratification			
Low	7 (87.5%)	13 (68.4%)	0.633
Intermediate	0 (0%)	6 (31.6%)	0.072
High	1 (12.5%)	0 (0%)	0.296
Grade 1/2 (n, %)	8 (100%)/0 (0%)	12 (63.2%)/7 (36.8%)	0.046
Initial treatment			
Watch-and-wait (n, %)	2 (40.0%)	1 (5.3%)	0.201
Chemotherapy (n, %)	3 (37.5%)	18 (94.7%)	0.004
Radiotherapy (n, %)	3 (37.5%)	0 (0%)	0.019
Clinical relapse (n, %)	1 (10.0%)	0 (0%)	0.370

FLIPI, Follicular Lymphoma International Prognostic Index; LDH, lactate dehydrogenase; and VCE, video capsule endoscopy.

**Table 3:** Detailed clinical course of seven patients with duodenal follicular lymphoma who relapsed, died, or were closely observed.

Patient	Age at DFL Diagnosis (Years)	Sex	Lugano Stage	VCE	Involved Site	Initial Treatment	Events (Time to Events)	Clinical Outcomes	Follow-Up Period
1	58	Male	Stage I	Yes	Duodenum second portion, multifocal small bowel	Watch-and-wait ^†^	No events	Alive with lymphoma	66.0 months
2	41	Male	Stage I	Yes	Duodenum second portion	Watch-and-wait	No events	Alive with lymphoma	26.7 months
3	48	Female	Stage I	Yes	Duodenum second portion	Watch-and-wait	No events	Alive with lymphoma	52.4 months
4	49	Female	Stage I	No	Duodenum second portion	Watch-and-wait	No events	Alive with lymphoma	30.6 months
5	43	Female	Stage II_2_E	No	Duodenum second portion Aortocaval and para-aortic LNs Pancreas	Rituximab monotherapy	Relapse (12.2 months)	Alive, CR after treatment BR as salvage regimen	58.3 months
6	77	Female	Stage I	No	Duodenum second portion	RT 24 Gy/12 Fx	AoV cancer (47.1 months)	Died due to AoV cancer	50.1 months
7	50	Female	Stage IV	Yes	Duodenum second portion Disseminated mesentery LNs	RT 24 Gy/12 Fx *	Relapse (10.1 months)	Alive, CR after treatment BR as salvage regimen	61.2 months

AoV, ampulla of the Vater; BR, bendamustine–rituximab; CR, complete response; DFL, duodenal follicular lymphoma; LN, lymph node; RT, radiotherapy; and VCE, video capsule endoscopy. ^†^ All the patients in the watch-and-wait category underwent regular endoscopic workup for 3–6 months until disease progression. * The majority of patients who were confirmed to have small bowel extension received systemic chemotherapy, with the exception of one patient who did not receive any treatment according to the patients’ desire. The patient refused to administer chemotherapy despite being diagnosed with stage IV DFL. Therefore, we decided to perform RT to observe the primary DFL site.

## Data Availability

The data supporting the findings of this study are available upon request from the corresponding author. The data are not publicly available because of privacy or ethical restrictions.

## Supplementary Materials

The following supporting information can be downloaded at: https://www.mdpi.com/article/10.3390/diagnostics15020193/s1, Table S1: Lugano classification for extranodal lymphoma and Table S2: Comparison of clinical characteristics and treatment modalities between localized and advanced-stage patients (N = 40). Figure S1. Various cases of duodenal-type follicular lymphoma involving distal jejunum.
