# Supplementary material for: The Role of Small Bowel Capsule Endoscopy in Determining the Treatment Strategy for Duodenal Follicular Lymphoma: A Single-Center Retrospective Study
Source: Diagnostics (Basel). 2025 Jan 15;15(2):193. doi: 10.3390/diagnostics15020193 (PMC11765495; doi:10.3390/diagnostics15020193)
Supplement: Supplementary file 1 [file diagnostics-15-00193-s001.zip › diagnostics-3377340-supplementary.pdf]

Table S1. Lugano classification for extranodal lymphoma

Classification	Features
**Stage I**	**Tumor confined to GI tract**<br>Single primary site or multiple, non-contiguous lesions
**Stage II**	**Tumor extending into abdomen from primary GI site**<br>Nodal involvement<br>II$_1$ local (para-gastric in cases of gastric lymphoma and para-intestinal for intestinal lymphoma)<br>II$_2$ distant (mesenteric in the case of an intestinal primary, otherwise; para-aortic, para-caval, pelvic, inguinal)
**Stage IIE**	**Penetration of serosa to involve adjacent organ or tissue**<br>Enumerate actual site of involvement, e.g. IIE$_{\text{[pancreas]}}$ IIE$_{\text{[large intestine]}}$ IIE$_{\text{[post-abdominal wall]}}$
**Stage IV**	**Disseminated extranodal involvement or a GI tract lesion with supra-diaphragmatic nodal involvement**

The subscript "E" originally denoted proximal or contiguous, extranodal disease that could be uncompressed within an irradiation field appropriate for nodal disease of the same anatomical extent. The concept of stage III disease within the context of GI tract lymphoma was deleted altogether, with supra-diaphragmatic nodal disease included within stage IV.

Table S2. Comparison of clinical characteristics and treatment modalities between localized and advanced-stage patients (N=40)

	Localized stage (N=23)	Advanced stage (N=17)	*P*-value
**Age >60 years**	3 (13.0%)	3 (17.6%)	1.000
**Male/female (n, %)**	10 (43.5%) / 13 (56.5%)	5 (29.4%) / 12 (70.6%)	0.364
**Hemoglobin <12.0 (g/dL)**	3 (13.0%)	4 (23.5%)	0.432
**Elevated LDH (U/L)**	2 (8.7%)	2 (11.8%)	1.000
**Nodal areas involvement >4**	0 (0%)	8 (47.1%)	<0.001
**Extranodal involvement ≥2**	0 (0%)	7 (41.2%)	0.001
**Bone marrow involvement**	0 (0%)	7 (41.2%)	0.001
**Lugano stage**			
**I**	22 (95.7%)	0 (0%)	<0.001
**II_1**	1 (4.3%)	0 (0%)	1.000
**II_2**	0 (0%)	4 (23.5%)	0.026
**IV**	0 (0%)	13 (76.5%)	<0.001
**FLIPI risk stratification**			
**Low**	23 (100%)	6 (35.3%)	<0.001
**Intermediate**	0 (0%)	9 (52.9%)	<0.001
**High**	0 (0%)	2 (11.8%)	0.174
**Grade 1/2 (n, %)**	20 (87.0%) / 3 (13.0%)	10 (58.8%) / 7 (41.2%)	0.042
**VCE performed**	15 (65.2%)	12 (70.6%)	0.720
**Small bowel involvement**	10 (66.7%)	9 (75.0%)	0.696
**Initial treatment**			
**Watch and wait (n, %)**	4 (17.4%)	0 (0%)	0.123
**Chemotherapy (n, %)**	10 (43.5%)	16 (94.1%)	0.001
**Radiotherapy (n, %)**	9 (39.1%)	1 (5.9%)	0.016
**Clinical relapse (n, %)**	0 (0%)	2 (11.8%)	0.174

FLIPI, Follicular Lymphoma International Prognostic Index; LDH, lactate dehydrogenase; VCE, video capsule endoscopy

Figure S1. Various cases of duodenal-type follicular lymphoma involving distal jejunum.

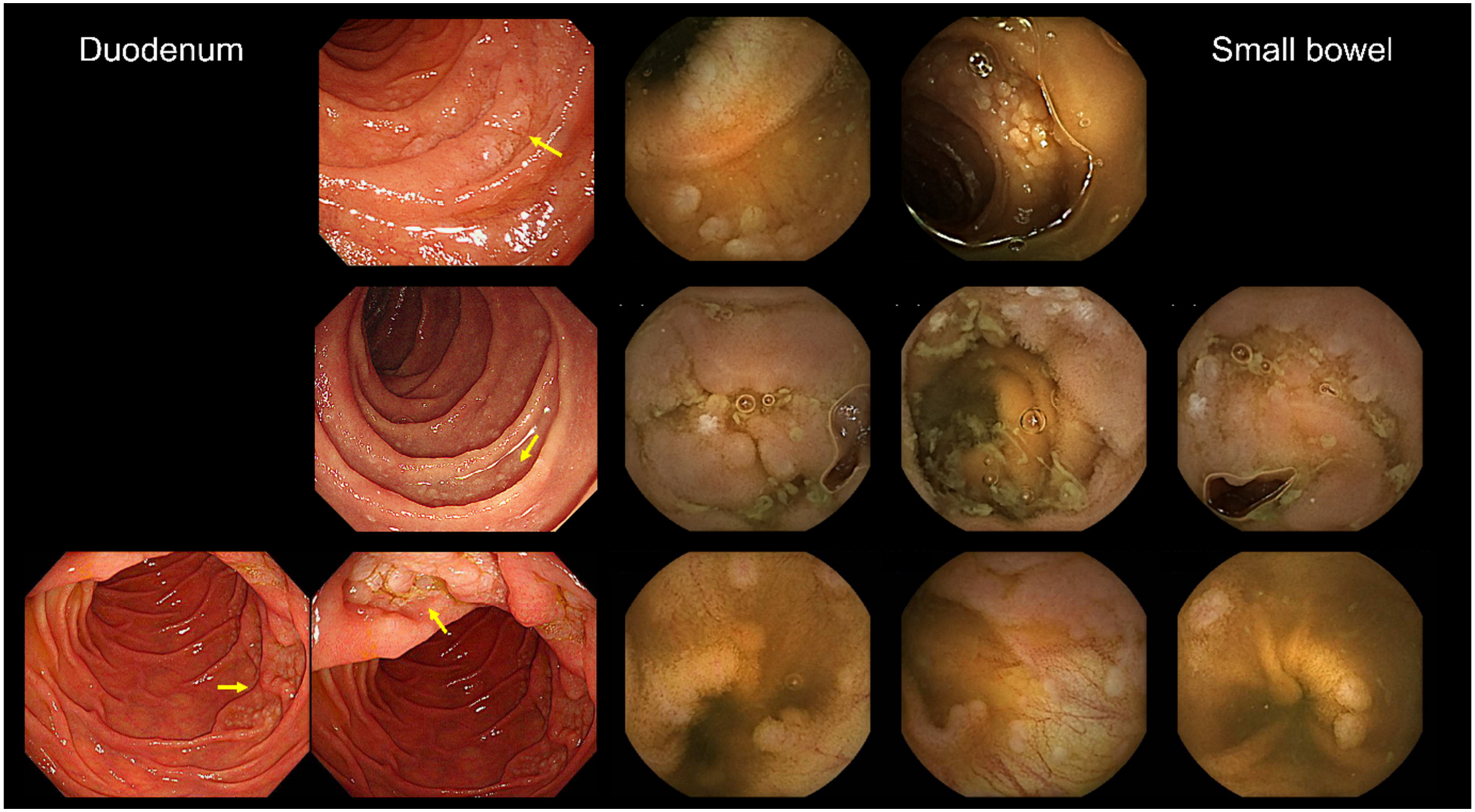

Left: Follicular lymphoma lesion in the duodenum, visualized through esophagogastroduodenoscopy. The yellow arrow indicates a mucosal lesion that was confirmed as duodenal-type follicular lymphoma through endoscopic biopsy.

Right: Follicular lymphoma lesions in the small bowel (jejunum and ileum), visualized through video capsule endoscopy.
